# Determination of *Helicobacter pylori* Virulence Genes in Gastric Biopsies by PCR

**DOI:** 10.1155/2013/606258

**Published:** 2013-04-15

**Authors:** Tamer Essawi, Wail Hammoudeh, Israr Sabri, Walid Sweidan, Mohammad A. Farraj

**Affiliations:** Master Program in Clinical Laboratory Science, Birzeit University, Birzeit 970, Palestine

## Abstract

*Aim*. The aim of this study was to identify the presence of *H. pylori* in biopsy specimens from symptomatic patients by PCR. In addition, the rate of *cagA*, *vacA*, *iceA1*, and *iceA2* virulence genes was determined. *Materials and Methods*. One hundred antral gastric biopsy specimens were collected during endoscopy from patients suffering from gastroduodenal symptoms. The samples were collected by the gastroenterologists in their own clinics in Ramallah, Palestine. DNA was extracted from the biopsies and subsequently used for PCR identification of *H. pylori* and the virulence genes using specific primers. *Results*. The rate of positive *H. pylori* in the collected biopsies was 44%. The rates of the virulence genes in this sample: *cagA*, *vacA*, *iceA1*, and *iceA2* were 65.9%, 40.9%, 63.6%, and 84.1%, respectively. *Conclusion*. The *iceA2* gene was the most frequent in this study. Much research is necessary to determine the presence of an association of this gene with gastric pathology. Variation in the rates of the *iceA* gene in different countries is a strong indication of its geographical distribution. This study would provide important information regarding the prevalence of virulence genes (*vacA*, *cagA*, *iceA1*, and *iceA2*) in *H. pylori* strains in the sample tested in this country.

## 1. Introduction

*Helicobacter pylori* is a microaerophilic, spiral shaped Gram-negative bacterium that colonizes the human stomach. It has been linked to chronic active gastritis, peptic ulcers disease, gastric cancer, and mucosa-associated lymphoid tissue lymphoma [1, 2]. *H. pylori* has been classified as a definite class I carcinogen by the World Health Organization [3]. Although the prevalence of *H. pylori *infection may exceed 70% in some developing countries [4, 5], only a small percentage of the population develop severe disease. This can be attributed to the involvement of specific factors that contribute to the pathogenicity of this organism. The cytotoxin-associated gene (*cagA*), a marker for cag pathogenicity island, is associated with severe clinical diseases as seen in peptic ulcer disease and gastric adenocarcinoma [1]. The vacuolating cytotoxin (*vacA*) gene encodes for the vacuolating cytotoxin, the pore forming toxin which causes progressive vacuolation and injury to gastric epithelium [6, 7]. The induced by contact with epithelium (*iceA*) A gene has been considered as the marker for peptic ulcer disease. 

The aims of this study were to identify *H. pylori* directly from biopsy specimen collected from symptomatic patients using primers to amplify the *ureA* and *glmM* (*ureC*) genes and to determine the rate of virulence genes, *cagA, vacA, iceA1* and *iceA2, *in the biopsy samples by PCR. 

## 2. Methods

### 2.1. Specimen Collection and Processing

Antral gastric biopsy specimens were collected during upper endoscopy from 100 patients suffering from gastroduodenal symptoms. Patient's consent to participate in this study was obtained prior to enrollment. The samples were collected by gastroenterologists between January and August 2012. DNA was extracted from the biopsies by the QIAamp DNA Mini Kit (Qiagen, Valencia, CA, USA) following the manufacturer's instructions. Extracted DNA was used for subsequent PCR experiments. 

### 2.2. Polymerase Chain Reaction

Amplification was conducted in a total volume of 25 *μ*L. The reaction mixture contained 12.5 uL, 2X ready PCR mix (Thermo Scientific) and consisted of 1.25 U Taq-Pol, 75 mM Tris-HCL (pH 8.8), 1.5 mM MgCl_2_, and 0.2 mM of each dNTP. The reaction mixture contained 12.5 uL master mix, 1.0 uM of each forward and reverse primers (Table 1), 1 ug DNA template, and 8.5 uL RNase free water to a total volume of 25 uL. The amplification was carried out in a C-1000 thermal cycler (Bio-Rad, USA) according to the following program: an initial denaturation step at 95°C for 10 min, followed by 35 cycles of denaturation at 95°C for 30 s, annealing, primer specific shown in Table 1 for 1 min, and a final extension step at 72°C for 5 min. Amplified PCR products were resolved by agarose gel electrophoresis (5 V/60 min) using 1,5% agarose in Tris Acetate-EDTA (TAE) buffer containing 0.5 ug/mL of ethidium bromide. Molecular size ladder of 100 bp (Fermentans, Germany) was used to determine the size of the bands. The gel was viewed and photographed on a Gel-Doc System (Bio-Rad, USA). The primers used for the amplifications were obtained from Invitrogen (Rhenium, Jerusalem), shown in Table 1. 

## 3. Results

Antral biopsies were collected from 100 patients. Molecular identification of *H. pylori* was performed on all biopsies by PCR using primers (HPU1, HPU2) to amplify a 411 bp product for the *ureA* gene and primers (GlmM-F, GlmM-R) to amplify a 294 bp product for the *glmM *(*ureC*) gene (Table 2). The rate of positive *H. pylori* in the biopsies tested was 44% (44/100). The rate of virulence genes *cagA, vacA, iceA1, *and *iceA2 *in the positive biopsies for *H. pylori* is summarized in Table 2. 

## 4. Discussion

The *glmM *gene is highly conserved and has been used to identify *H. pylori* in gastric biopsies. Although it has been reported that the sensitivity and specificity of *ureA* is more than 90% [8], the *glmM *gene has better sensitivity than the *ureA* gene [9]. One of the advantages of using the *glmM* gene to identify *H. pylori* directly in gastric biopsies is its high degree of sensitivity and specificity, since it has a detection rate of 10 to 100 *H. pylori* cells which is better than histopathology [10]. Our findings revealed a rate of positive *H. pylori* in the tested biopsies of 44% based on direct molecular detection by PCR using the *ureA* and the *glmM* genes. To improve DNA extraction from the biopsies and to eliminate PCR inhibitors, a special extraction kit from Qiagen was used [11]. 

The induced by contact with epithelium (*iceA*) gene has two allelic forms, *iceA1* and *iceA2.* The *iceA1* gene is expressed by *H. pylori* upon contact with the gastric epithelial cells [12]. Although *iceA *gene has not been associated with gastric cancer, there is an unresolved controversy for the role of this gene in gastric pathology. Reports have associated the *iceA1* allele in peptic ulcer [12, 13] while others did not find any role for this allele in gastroduodenal disease [13]. The *iceA2* allele has been inversely associated with peptic ulcer [14].

There are significant variations reported regarding the prevalence of if *iceA1* and *iceA2 *alleles. The iceA1 has been reported to be the prevalent allele in some studies [12] while iceA2 the prevalent allele in others [15].

The *iceA2* gene was the most frequent in this study (84.4%). A Brazilian study reported a rate of 90.1% for the *iceA2* allele [16]. Contrary to that, a Mexican study reported a rate of 9% for the *iceA2 *allele, while 72% carried both genes *iceA1* and *iceA2 *[17]. In East Asia, the *iceA1* genotype has been reported to be the predominant (76%) while in Portugal and Colombia *icaA2* is predominant [18]. The rate of the *iceA1* gene in this study was 62.2% and 53.3% of the samples carried both genes *iceA1* and *iceA2*. This variation in rates for the *iceA *gene in different countries is a strong indication of its geographical distribution. 

The *cagA* results in our study of 65.9% are similar to those obtained in Tunisia of 61.6% [19]. They are higher than those obtained in Pakistan (56%), but lower than rates reported in Iran (76%), Iraq (71%) [19], India, and Bangladesh of 70% [20]. The cagA positive strains in the Mexican study was 86% [17]. In Japan, the rate of *cagA* is very high (90%) [19], which is correlated with the commonly encountered gastric cancer in that country. 

The *cagA* and the *vacA *genes are major virulence factors in *H. pylori *responsible for the gastric pathology. The polymorphic nature of the *vacA* gene, due to allelic variations in the signal and middle regions of the gene, has not been addressed in this study. The rate of the *vacA* gene in the sample tested was 40.9%. *vacA* negative *H. pylori* has been detected in biopsy specimens in Sweden [21]. Our findings revealed the presence of 7 combinations of genotypes based on the *cag/vac* genes as shown in Table 2. In one biopsy specimen, *cagA* negative/*vacA* negative genotype was encountered. Although the other virulence genes were tested (*iceA1* and *iceA2*), the identity of this isolate as *H. pylori* was confirmed by repeating the amplification with *ureA* primers.

In conclusion, this study would provide important information regarding the rate of virulence factors in this country. Determination of virulence genes may provide information regarding the risk of clinical outcomes in symptomatic patients.

## Figures and Tables

**Table 1:** Primers used for the amplification of *H. pylori* genes.

Primer	5′-3′ sequence	Product size (bp)	Annealing (°C)
CagA-F	AATACACCAACGCCTCCAAG	400	55
CagA-R	TTGTTGGCGCTTGCTCTC		

VacA-3.AS	GCCGATATGCAAATGAGCCGC	678	66
VacA-1.SE	CAATCGTGTGGGTTCTGGAGC		

IceA1-F	CGTTGGGTAAGCGTTACAGAATTT	558	56
IceA1-R	TCATTGTATATCCTATCATTACAAG		

IceA2-F	GTTGTCGTTGTTTTAATGAA	120	50
IceA2-R	GTCTTAAACCCCACGATTAAA		

HPU1	GCCAATGGTAAATTAGTT	411	45
HPU2	CTCCTTAATTGTTTTTAC		

GlmM-F	AAGCTTTTAGGGGTGTTAGGGGTTT	294	55
GlmM-R	AAGCTTACTTTCTAACACTAACGC		

**Table 2:** Genes used in the PCR identification of *H. pylori* and virulence genes determination.

	Genes					
	*cagA *	*vacA *	*iceA1 *	*iceA2 *	*ureA *	*gl* *mM* (*ureC*)
Positive	29 (65.9%)	19 (43.2%)	28 (63.6%)	37 (84.1%)	41 (93.2%)	43 (98%)
Negative	15 (34.1%)	25 (56.8%)	16 (36.4%)	7 (15.9%)	3 (6.8%)	1 (2%)

Total	44 (100%)	44 (100%)	44 (100%)	44 (100%)	44 (100%)	44 (100%)
